# Characterization of the complete mitochondrial genome of *Onychostoma macrolepis* (Teleostei: Cyprinidae)

**DOI:** 10.1080/23802359.2019.1677190

**Published:** 2019-10-12

**Authors:** Xiaoting Gong, Chao Meng, Hao Xu, Guanghui Tian, Yu Sun, Huichao Lu

**Affiliations:** College of Life Sciences, Shandong First Medical University & Shandong Academy of Medical Sciences, Taian, China

**Keywords:** *Onychostoma macrolepis*, mitochondrial genome, phylogenetic tree

## Abstract

In this study, the entire mitogenome sequence of the *O. macrolepis* has been sequenced. However, its systematic classification is still undetermined. The complete mitochondrial genome is 16,621bp, which includes 37 genes (13 protein-coding genes, 2 rRNA genes and 22 tRNA genes) and 1 control region.The overall base composition is 34.52% A, 19.01% T, 25.58% C, 20.89% G, showing AT rich feature (55.76%). Its structure type is similar to the mitogenome of Cyprinidae. Phylogenetic tree showed that *O. macrolepis* belong to Barbinae.

*Onychostoma macrolepis*, is a genus of Cyprinid that distributes the freshwater in Culai, China. Due to the climate change and overfishing, the wild population of the fish is declining. The golden fish are even rarer. We systematically sequenced and assembled the complete mitochondrial genome of the fish.

Samples were collected from the lake in Mountain Culai (36.03 N/117.22E) and frozen in –80 °C. Voucher specimen (SL2017006) was deposited in Specimens Laboratory, Shandong First Medical University.The Genomic DNA was extracted from muscle tissue using standard phenol-chloroform method. Primers were designed according to conservative region of relative species. The PCR products were sequenced by Sanger sequencing and then assembled by BioEdit 3.0 (Hall 1999). The *O. macrolepis* mitogenome had been deposited in GenBank (accession number: MH998249). The total length of mitochondrial genome is 16,621 bp. The rates of A, T, C, G were 34.52%, 19.01%, 25.58%, 20.89%. The content indicated an obvious bias towards AT(55.8%). Similar to other previously Cyprinid fish, the mitogenome of *O. macrolepis* also consisted of the typical 37 genes(13 protein coding genes, 22tRNAs and 2rRNAs) and 1 non-coding control region(*D-loop*) (Luo et al. 2018; Ye et al. 2018). Except the *ND6* and 8tRNA genes (*tRNA-Ala, tRNA-Asn, tRNA-Tyr, tRNA-Gln, tRNAPro, tRNA-Glu, tRNA-Cys,* and *tRNA-Ser*), all other genes were encoded on the H-strand. All genes were similar as in other bony fishes (Boore 1999). The rRNA genes are 956 and 1675 bp, separated by the *tRNA-Val* gene, located between *tRNA-Leu* and *tRNA-Phe*. The tRNA genes range in size from 67 to 76 bp. The non-coding region between the *tRNA-Pro* and *tRNA-Phe* genes was considered as the control region which is 965 bp.

Except that *COX1* uses GTG as the start codon, the other 12 protein-coding genes started with an ATG codon. All PCGs harbour the typical TAA or TAG stop codons; except the *ND2*, *COX2*, *NAD3*, *ND4* and *CYTB* with incomplete stop codon T(aa). The total length of all encoded protein genes in *O. macrolepis*is 10,269 bp. Among the 13 protein genes, there are three overlapping regions, in which there are 7 bp shared bases between *ATP8*/*ATP6* and *ND4L/ND4*, and *ND5/ND6* has 4 bp base overlap.

The phylogenetic position of the *O. macrolepis* was determined by neighbour joining (NJ) methods to reconstruct the phylogenetic tree, implemented in MEGA7.0 (Kumar et al. 2016) (Figure 1). Based on the analysis of the whole mitochondrial genome of 18 species, the phylogenetic tree indicated that *O. macrolepis* had the closet relationship with *O. lini*, which clustered into a clade with other Onychostoma. It is obvious from the evolutionary tree that *O. macrolepis* belong to Barbinae and have distinct branches with other families. As is shown, Onychostoma and Acrossocheilus were positioned as a sister genus. As expected, the obtained mitochondrial genome resources will provide valuable molecular information for future protective studies.

**Figure 1.**
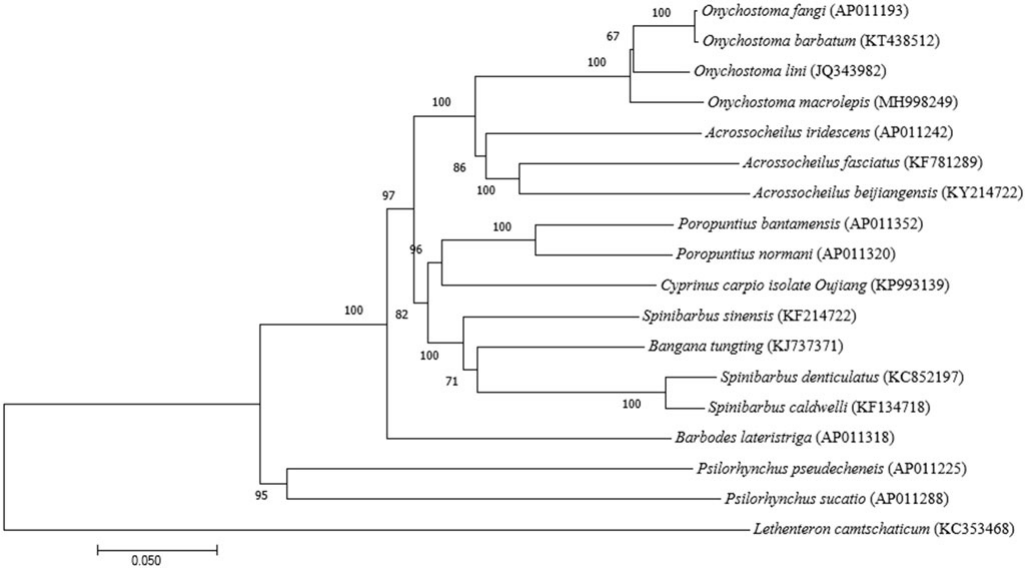
Neighbor Joining phylogenetic tree generated by alignment of complete mitogenome sequences of 18 species. The bootstrap values are indicated 1000 resamplings.
